# Manganese Utilization in *Salmonella* Pathogenesis: Beyond the Canonical Antioxidant Response

**DOI:** 10.3389/fcell.2022.924925

**Published:** 2022-07-12

**Authors:** Siva R. Uppalapati, Andres Vazquez-Torres

**Affiliations:** ^1^ Department of Immunology & Microbiology, University of Colorado School of Medicine, Aurora, CO, United States; ^2^ Veterans Affairs Eastern Colorado Health Care System, Denver, CO, United States

**Keywords:** manganese, *Salmonella*, virulence, mismetallation, carbon metabolism, central metabolism, oxidative stress, nitrosative stress

## Abstract

The metal ion manganese (Mn^2+^) is equally coveted by hosts and bacterial pathogens. The host restricts Mn^2+^ in the gastrointestinal tract and *Salmonella-*containing vacuoles, as part of a process generally known as nutritional immunity. *Salmonella enterica* serovar Typhimurium counteract Mn^2+^ limitation using a plethora of metal importers, whose expression is under elaborate transcriptional and posttranscriptional control. Mn^2+^ serves as cofactor for a variety of enzymes involved in antioxidant defense or central metabolism. Because of its thermodynamic stability and low reactivity, bacterial pathogens may favor Mn^2+^-cofactored metalloenzymes during periods of oxidative stress. This divalent metal catalyzes metabolic flow through lower glycolysis, reductive tricarboxylic acid and the pentose phosphate pathway, thereby providing energetic, redox and biosynthetic outputs associated with the resistance of *Salmonella* to reactive oxygen species generated in the respiratory burst of professional phagocytic cells. Combined, the oxyradical-detoxifying properties of Mn^2+^ together with the ability of this divalent metal cation to support central metabolism help *Salmonella* colonize the mammalian gut and establish systemic infections.

## Introduction

A rich microbiome, cellular and abiotic mucosal barriers, as well as cellular and humoral effectors of the innate and adaptive immune system may limit the colonization, growth and spread of pathogenic bacteria in mammalian hosts (Rosales and Uribe-Querol, 2017; Levinson et al., 2018; Uribe-Querol and Rosales, 2020). Hosts use metal transporters, calprotectins, siderocalins and a variety of other metal-sequestering proteins to limit the availability of Mg^2+^, Fe^2+^, Mn^2+^ and Zn^2+^ ions from bacteria, a phenomenon known as nutritional immunity (Bellamy, 2003; Loomis et al., 2014; Hennigar and McClung, 2016; Wang et al., 2018; Cunrath and Bumann, 2019; Monteith and Skaar, 2021). Bacteria display sophisticated transport systems that counteract metal restrictions imposed by hosts (Porcheron et al., 2013; Chandrangsu et al., 2017). Among the bioactive metal ions, Mn^2+^ plays a salient role in bacterial physiology and the adaptation of prokaryotic cells to stress (Papp-Wallace and Maguire, 2006). Mn^2+^ is a cofactor of enzymes involved in carbon and nucleotide metabolism, DNA replication, and protein translation (Lovley and Phillips, 1988; Martin and Imlay, 2011; Culotta and Daly, 2013; Torrents, 2014; Kaur et al., 2017; Tong et al., 2017; Daniel et al., 2018; Li and Yang, 2018; Hutfilz et al., 2019). Mn^2+^ is also a cofactor of superoxide dismutase (SOD) and catalase (KatN) family members, and this divalent metal is utilized by carbon utilization enzymes such as phosphoglycerate mutase (PGM) and fructose-1,6-bisphosphate phosphatase, or envelope stress phosphatases (Shi et al., 2001; Miriyala et al., 2012; Whittaker, 2012; Radin et al., 2019). The MntR repressor coordinates a Mn^2+^ ion and the ferric uptake regulator (Fur) can bind to Fe^2+^ or Mn^2+^ (Hiemstra et al., 1999; Kehres et al., 2002a; Ikeda et al., 2005; Waters, 2020).

*Salmonella enterica* serovar Typhimurium is a facultative intracellular pathogen associated with intestinal and invasive diseases in humans and animals (Galan, 2021). The success of *Salmonella* as a pathogen can be directly ascribed to its capacity to overcome colonization resistance by resident gut microbiota, invade epithelial cells, and establish intracellular infections within host enterocytes and macrophages (Winter and Bäumler, 2011; Lorkowski et al., 2014; Lhocine et al., 2015; Aljahdali et al., 2020; Jiang et al., 2021; Powers et al., 2021). During their associations with host cells, *Salmonella* are exposed to acid pH, oxidative and nitrosative stress, and nutritional deprivation (Fang et al., 2016). A variety of adaptive mechanisms are used by *Salmonella* to fight the hostile host environment (Bernal-Bayard and Ramos-Morales, 2018; Tanner and Kingsley, 2018; Gogoi et al., 2019), and a few reviews have examined the contribution of Mn^2+^ in *Salmonella* pathogenesis (Kehres and Maguire, 2003; Papp-Wallace and Maguire, 2006; Osman and Cavet, 2011). Despite these advances, many unanswered questions and occasional contradictory findings warrant a review of our current understanding of Mn^2+^-mediated stress responses in *Salmonella* pathogenesis. In this review, we discuss the adaptive mechanisms that allow *Salmonella* to compete with the host for Mn^2+^, and present ways by which this divalent metal contributes to metabolic programs associated with resistance of *Salmonella* to oxidative and nitrosative stress.

## Manganese Limitation in the Host

Hosts sequester transition metals in their fight against pathogenic organisms (Hennigar and McClung, 2016). Metal sequestration is mediated by calprotectin, lipochalin 2, metallothioneins, ferritin and transport systems including NRAMP1. Mn^2+^ limitation is mostly mediated by calprotectin and NRAMP1.

### Calprotectin

The host protein calprotectin, a member of the calcium-binding S100 family, limits Mn^2+^ from extracellular *Salmonella*. Human calprotectin is a heterooligomer with a dedicated Zn^2+^-binding site-1 and a versatile Mn^2+^-, Fe^2+^-, Zn^2+^- and Ni^2+^-binding site-2 (Hayden et al., 2013; Zygiel and Nolan, 2018). Calprotectin is secreted by infiltrating neutrophils at sites of inflammation, reaching extracellular concentrations of about 40 μM, but is also expressed by epithelial cells and keratinocytes (Johne et al., 1997; Zygiel and Nolan, 2018; Jukic et al., 2021). Calprotectin limits metal bioavailability from bacteria, thus inhibiting bacterial growth*,* and its expression in epithelial cells diminishes binding of *Salmonella* to host cells (Nisapakultorn et al., 2001). Infection of gut mucosa by *Salmonella* attracts neutrophils, which secrete calprotectin in extracellular traps (Urban et al., 2009; Patel and McCormick, 2014). Fecal calprotectin levels in diarrheic children correlate with the severity of bacterial infection, and the concentration of calprotectin is increased in plasma during acute salmonellosis (Chen et al., 2012; De Jong et al., 2015). The acidic pH typical of infectious sites disrupts the tetramerization of calprotectin, not only attenuating its capacity to bind Mn^2+^ but also impairing its growth-inhibiting properties (Menkin, 1956; Rosen and Nolan, 2020). Although calprotectin inhibits *Salmonella* growth *in vitro*, its effectiveness in the intestinal lumen is severely limited by *Salmonella*’s Zn^2+^ and Mn^2+^ metal transporters (Liu et al., 2012; De Jong et al., 2015; Diaz-Ochoa et al., 2016).

### NRAMP1

The integral membrane protein NRAMP1, which is also known as SLC11A1, transports divalent transition metals such as Fe^2+^, Mn^2+^, Co^2+^ and Mg^2+^ (Canonne-Hergaux et al., 1999; Forbes and Gros, 2003; Cellier et al., 2007; Cunrath and Bumann, 2019). The expression of NRAMP1 is restricted to lysosomal compartments of monocytes and macrophages, whereas the highly homologous NRAMP2 protein is widely distributed on most cells (Vidal et al., 1995; Gunshin et al., 1997). The expression of NRAMP1 is maximal at late stages in the maturation of phagosomes (Gruenheid et al., 1997). NRAMP1 can import or efflux metals into the phagosome (Atkinson and Barton, 1999; Kuhn et al., 1999; Zwilling et al., 1999; Jabado et al., 2000), although recent structural studies have suggested a strong unidirectional efflux movement under physiological conditions (Bozzi and Gaudet, 2021). The directionality of NRAMP1-mediated ion transport is dependent on pH (Goswami et al., 2001). Although the affinity of human or mouse NRAMP1 to Mn^2+^ has not been determined, a homologous transporter from *Arabidopsis* has a K_m_ of 28 nM, (i.e., about 4-fold higher than the affinity of the *Salmonella* transporters MntH and SitABCD for Mn^2+^) (Kehres et al., 2000; Kehres et al., 2002b; Cailliatte et al., 2010).

Nutritional immunity imposed by NRAMP1 is a significant defense determinant against *Salmonella* as vividly illustrated by the high resistance of Sv129S6 or C3H/HeN mice carrying an intact *nramp1* locus and the propensity of BALB/c or C57BL/6 mice bearing the mutant allele *nramp1G169D* to develop severe *Salmonella* infections (Brown et al., 2013; Wendy P.; Loomis et al., 2014). Recent work argued that NRAMP1 contributes to *Salmonella* pathogenesis by depriving phagosomes of Mg^2+^ (Cunrath and Bumann, 2019). However, the latter model appears to be in conflict with the observation that the attenuation of *Salmonella* bearing mutations in the Mn^2+^ transport system SitABCD or the Mn^2+^-dependent SpoT enzyme are contingent on the expression of a functional NRAMP1 (see below). More investigations are needed to definitively identify the metal specificity of NRAMP1 in different tissues and different times in the course of the *Salmonella* infection.

## Mn^2+^ Import Promotes *Salmonella* Pathogenesis

**FIGURE 1:**
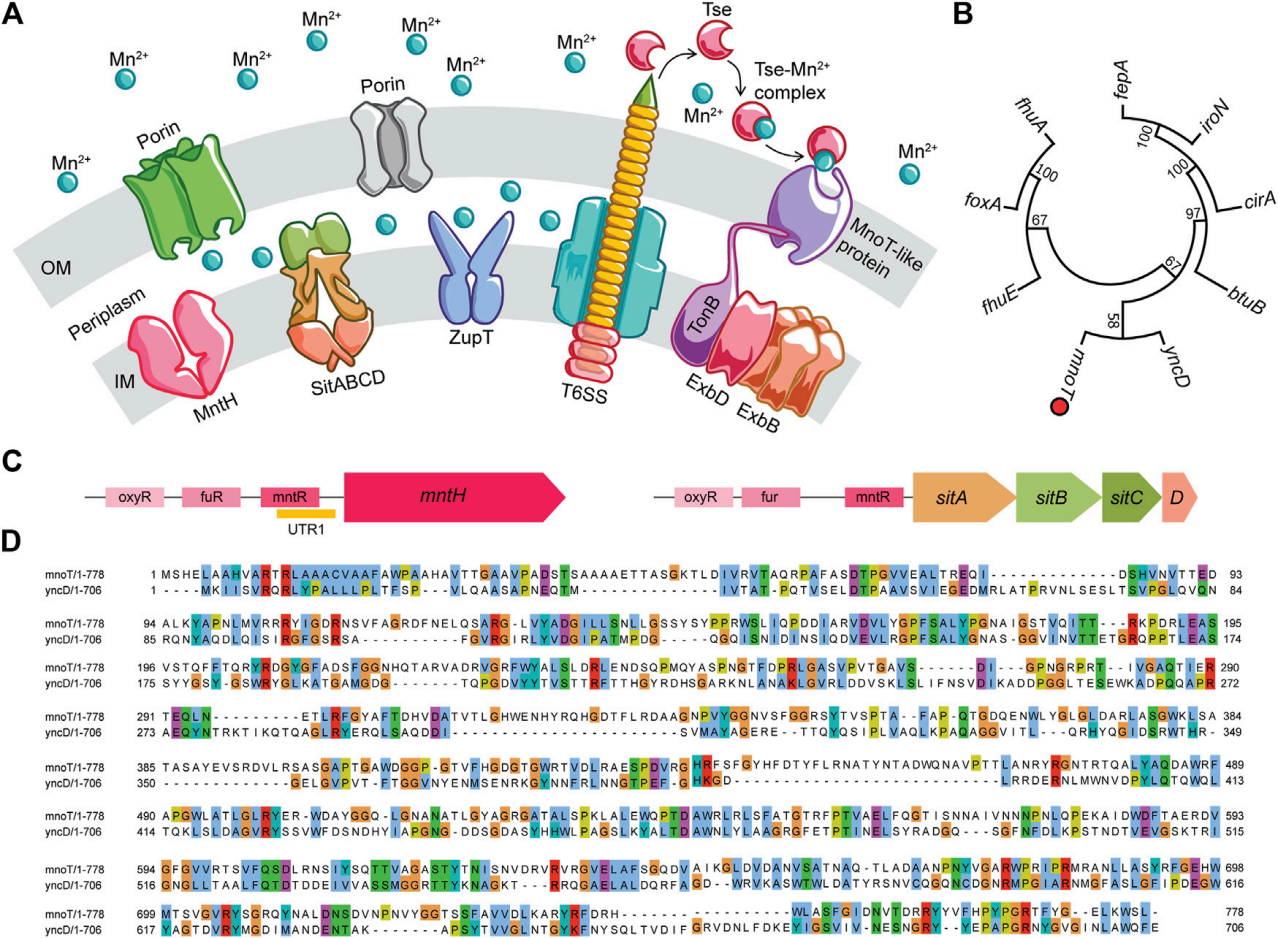
Manganese transport systems in *Salmonella*. **(A)** While the outer membrane is permeable to Mn^2+^ ions, the inner membrane imports this metal ion via specific and non-specific transporters like MntH, SitABCD and ZupT. A type VI secretion system dependent Mn^2+^ acquisition mechanism observed in pathogens such as *Burkholderia*, *Vibrio*, and *Yersinia* is proposed. **(B)** Neighbor-joining tree of MnoT-like proteins identified in *Salmonella* genome by Pattern Hit Initiated BLAST. **(C)** Genetic organization of *mnTH* and *sitABCD* operons with regulatory elements in the promoter regions. **(D)** Clustal alignment of *Burkholderia* MnoT (WP_171466016) and *Salmonella* YncD (ACY88391.1) proteins by Clustal Omega aligner. The alignment results were extracted and reformatted in MView command line utility. All protein identities were normalized by aligned length and the residues are colored using the default built-in colormap.

Entry of Mn^2+^ into the periplasmic space is mostly mediated by non-specific porins on the outer membrane, whereas transporters in the cytoplasmic membrane actively influx this divalent metal cation into the bacterial cytoplasm (Figure 1A). MntH, a proton-dependent NRAMP1 homolog, actively imports Mn^2+^ with a K_*m*_ of 0.1 μM (Kehres et al., 2000). *Salmonella* upregulate MntH expression in response to both low Mn^2+^ concentrations and oxidative stress (Kehres et al., 2002a; Cunrath and Palmer, 2021). MntH mutant *Salmonella* grow poorly in mouse macrophages, but replicate rather well in mice (Boyer et al., 2002; Zaharik et al., 2004; Diaz-Ochoa et al., 2016). The slight attenuation of *mntH* deficient *Salmonella* suggests the existence of redundant transport systems. Genetic analyses revealed the presence of a Mn^2+^ responsive element in the promoter of the *sitABCD* operon that is encoded within the *Salmonella* pathogenicity island-1 gene cluster (Janakiraman and Slauch, 2000; Kehres et al., 2002b). The SitABCD transporter is a typical ABC transporter consisting of a periplasmic-binding protein SitA, an ATP-binding protein SitB and two integral membrane permeases, SitC and SitD. Initially, the SitABCD transporter was thought to function as a Fe^2+^ transport system, as this locus is regulated by Fur under iron-limiting conditions (Janakiraman and Slauch, 2000; Ikeda et al., 2005). However, metal ion uptake studies have shown that SitABCD mediates influx of Mn^2+^ with an apparent affinity of 0.1 μM, transporting Fe^2+^ with 30–100 times lower efficiency (Kehres et al., 2002b). A third transporter with broad cation specificity, ZupT, has also been implicated in Mn^2+^ transport in *Salmonella* during nitrosative stress (Yousuf et al., 2020).

Low Mn^2+^ concentrations activate the expression of MntH and SitABCD (Kehres and Maguire, 2003; Chandrangsu et al., 2017; Bosma et al., 2021). The *mntH* gene is repressed by both MntR and Fur in response to high Mn^2+^ and iron replete conditions, respectively (Kehres et al., 2000; Kehres et al., 2002a; Troxell et al., 2011; Powers et al., 2021). Expression of *mntH* is also induced by peroxide via H_2_O_2_-sensing OxyR regulatory protein (Kehres et al., 2002a) (Figure 1C). The persistence of Mn^2+^-dependent repression of *mntH* in Δ*mntR Salmonella* points to the existence of MntR-independent control. Analysis of the *mntH* 5′ UTR identified an Mn^2+^-sensing riboswitch that forms a Rho-independent terminator (Shi et al., 2014; Scull et al., 2020) (Figure 1C). The presence of several transcriptional breakpoints suggests dynamic expression of MntH in diverse host niches. MntR, Fur and OxyR elements are also present in the *sitABCD* promoter (Ikeda et al., 2005). The *sitABCD* operon does not contain, however, a riboswitch-like sequence at the 5′ UTR. Despite the similarities in transcriptional control and their identical affinity for Mn^2+^, MntH and SitABCD have specialized functions. MntH primarily transports Mn^2+^ at acidic pH, whereas SitABCD preferentially works at alkaline pH (Kehres et al., 2002b), suggesting that *Salmonella* may preferentially utilize MntH or SitABCD at different anatomical locations or times during infection (Ikeda et al., 2005).

*mntH* mutant *Salmonella* display little attenuation, whereas *sitABCD* mutants are attenuated in systemic models of infection (Janakiraman and Slauch, 2000; Boyer et al., 2002; Zaharik et al., 2004). Attenuation of *sitABCD* mutant *Salmonella* is contingent on the presence of host NRAMP1 (W. P. Loomis et al., 2014; Zaharik et al., 2004). Additionally, the non-specific transporter ZupT also competes with the host NRAMP1 for Mn^2+^ metal ions in phagosomes, contributing to *Salmonella* virulence (Karlinsey et al., 2010). By competing for Mn^2+^ with host cell calprotectin, MntH and SitABCD Mn^2+^ transport systems help *Salmonella* overgrow commensals in the inflamed gut (Diaz-Ochoa et al., 2016). Acquisition of Mn^2+^ also powers detoxification of ROS produced by inflammatory neutrophils in the gut mucosa (Diaz-Ochoa et al., 2016). The role of MntH and SitABCD may not be limited to extracellular bacteria, as genes encoding these Mn^2+^ transporters are transcribed in *Salmonella* residing in the cytosol of epithelial cells, and *mntH* and *sitA Salmonella* mutants grow poorly in the cytosol of epithelial cells (Powers et al., 2021). The acquisition of Mn^2+^ by cytosolic *Salmonella* may counteract oxidative stress, while facilitating utilization of sugars (Powers et al., 2021).

*Salmonella* deficient of MntH, SitA and ZupT transporters still acquire trace levels of Mn^2+^ *in vitro*, indicating the presence of other import systems (Karlinsey et al., 2010; Yousuf et al., 2020). The search for other modes of Mn^2+^ import into *Salmonella* is still underway, and examples from other bacteria may lead to the discovery of novel Mn^2+^ uptake systems in *Salmonella*. *Burkholderia pseudomallei* import Mn^2+^ via a type VI secretion system (T6SS) and the TonB cell envelope protein (Si et al., 2017; DeShazer, 2019). *B. pseudomallei* undergoing oxidative stress secrete the Mn^2+^-binding T6SS effector TseM, and low Mn^2+^ concentrations induce expression of the Mn^2+^ specific, TonB-dependent MnoT integral protein (Figure 1A). TseM scavenges extracellular Mn^2+^ and actively shuttles the metal via MnoT. *Salmonella* T6SS is activated by oxidative stress and our bioinformatics analysis has revealed a conserved locus in the *Salmonella* genome with high similarity to *Burkholderia* MnoT (Figures 1B,D) (Kroger et al., 2013). Additional T6SS substrates with Mn^2+^-scavenging capacities are still being uncovered, including the TssS micropeptide from *Yersinia pseudotuberculosis* that chelates Mn^2+^ and sabotages bacterial clearance by inhibiting STING-mediated innate immune response (Zhu et al., 2021).

## Mn^2+^ Export in *Salmonella* Pathogenesis

Paradoxically, excessive Mn^2+^ evokes oxidative stress in *E. coli*, affecting protein stability, interfering with envelope biogenesis, disrupting iron homeostasis and diminishing both tricarboxylic acid cycle and electron transport chain functions (Kaur et al., 2017). Bacteria excrete excessive Mn^2+^ using both the LysE superfamily MntP protein, and the cation diffuser facilitator (CDF) family member MntE (Martin et al., 2015). The *Xanthomonas* MntP homolog has two DUF204 domains that are conserved in *Salmonella* MntP protein (Li et al., 2011). MntP is regulated at transcriptional and posttranscriptional levels via MntR, mismetallated Fur and a *yybP*-*ykoY* riboswitch (Dambach et al., 2015; Bosma et al., 2021). The expression of the small RNA *rybA,* which encodes the small protein MntS, is repressed by MntR (Waters et al., 2011; Martin et al., 2015). The accumulation of apo-MntR in Mn^2+^ starving cells activates production of MntS, which represses MntP efflux activity and thus enlarges the intracytoplasmic pool of Mn^2+^ (Martin et al., 2015). On the other hand, the *yybP-ykoY* riboswitch directly binds to Mn^2+^, stabilizing a secondary structure that prevents sequestration of the *mntP* ribosome-binding site during translation (Dambach et al., 2015). MntP mediates efflux of Mn^2+^ ions in *Salmonella* following nitrosative stress (Ouyang et al., 2022).

The second efflux pump MntE is widely distributed in Gram-positive bacteria (Chandrangsu et al., 2017; Lam et al., 2020). *Streptococci* bearing mutations in *mntE* harbor excessive intracellular Mn^2+^ and experience attenuation of virulence (Rosch et al., 2009). Our bioinformatic analysis shows that *Salmonella* FieF (Yiip) protein belonging to CDF family has around 26%–64% sequence similarity with *Streptococcus* MntE, although it mediates zinc and iron export (Grass et al., 2005; Huang et al., 2017).

## Mn^2+^ Helps *Salmonella* Adapt to Oxidative Stress

*Salmonella* are exposed to ROS generated in the innate host response. Sulfur-containing cysteine and methionine amino acids are primary targets of H_2_O_2_ (Bin et al., 2017). In addition, ROS carbonylate arginine, lysine, proline and threonine residues, and oxidize metal prosthetic groups and histidine residues (Ortiz de Orue Lucana et al., 2012; Chang et al., 2020). *Salmonella* have evolved diverse mechanisms to counter ROS generation, prevent formation of hydroxy radicals, inhibit delivery of ROS into *Salmonella*-containing vesicles, detoxify and scavenge ROS, or repair the resultant protein and DNA modifications (Buchmeier et al., 1995; De Groote et al., 1997; Vazquez-Torres et al., 2000a; Vazquez-Torres et al., 2000b; Gallois et al., 2001; Vazquez-Torres and Fang, 2001; Waterman and Holden, 2003; Halsey et al., 2004; Aussel et al., 2011; Bogomolnaya et al., 2013; Song et al., 2013; Rhen, 2019; Bogomolnaya et al., 2020; Shome et al., 2020). Of particular interest to this review, Mn^2+^ protects *Salmonella* from ROS-mediated cytotoxicity by serving as a cofactor for SOD and KatN enzymes, replacing Fe^2+^ in the active sites of mononuclear iron-containing enzymes, and acting as a nonproteinaceous antioxidant (Culotta and Daly, 2013; Imlay, 2014; Ighodaro and Akinloye, 2018).

### Manganese-Based Detoxification of ROS

*Salmonella* confronts exogenous ROS generated by either host NADPH oxidase in phagocytes or dual oxidase 2 in epithelial cells (Vazquez-Torres and Fang, 2001; Behnsen et al., 2014). Superoxide anion (O_2_^.-^) formed by the vectorial transfer of electrons from flavoproteins and semiquinones to molecular oxygen is also a source of endogenous oxidative stress (Imlay, 2013). SODs and catalases expressed basally scavenge endogenously produced O_2_•^-^ and H_2_O_2_, which accumulate at steady-intracellular concentrations of ∼0.2 and ∼50 nM, respectively (Imlay, 2013). Cytoplasmic membranes are semipermeable to exogenous H_2_O_2_, but at neutral pH prevent entry of O_2_•^-^ (Bienert et al., 2006; Imlay, 2019). However, the HO_2_• acid conjugate readily reaches the bacterial cytoplasm (Imlay, 2019). *Salmonella* synthesizes two structurally distinct classes of SOD enzymes that catalyze the disproportionation of O_2_•^-^ to O_2_ and H_2_O_2_ (Perry et al., 2010; Rhen, 2019). Both Mn and Fe-dependent SODs (SodA and SodB, respectively) are cytoplasmic, whereas Cu,Zn-dependent SodC-I and SodC-II are periplasmic (Tsolis et al., 1995; Canvin et al., 1996; Taylor et al., 2009; Perry et al., 2010; Bismuth et al., 2021). Cu,Zn-SOD protect periplasmic or inner membrane targets from O_2_•^-^ toxicity, and limit peroxynitrite formation from the reaction of O_2_•^-^ and nitric oxide (NO•) (De Groote et al., 1997; Gort et al., 1999). Mutants devoid of cytoplasmic Mn-SOD and Fe-SOD are auxotrophic for branched chain amino acids, sulfur-containing amino acids, and aromatic amino acids, and, due to defects in aconitase and fumarase, can only grow on fermentable carbon sources (Carlioz and Touati, 1986; Imlay and Fridovich, 1992). *Salmonella* lacking Mn-SOD are susceptible to early killing by J774 macrophages but are virulent in an acute mouse model of *Salmonella* infection, likely reflecting the existence of redundant antioxidant systems (Tsolis et al., 1995). However, a *Salmonella* strain deficient in Mn-SOD is at a competitive disadvantage in the gut because of the Mn^2+^ limitation imposed by calprotectin (Diaz-Ochoa et al., 2016). Collectively, these investigations indicate that the role played by Mn-SOD in *Salmonella* pathogenesis is tissue specific.

*Salmonella* degrade H_2_O_2_ with the aid of the catalase activity of KatG, KatE, and KatN, of which KatN uses Mn^2+^ as cofactor. H_2_O_2_ induces transcription of *katG* in an OxyR-dependent manner, whereas *katE* and *katN* are members of the RpoS regulon (Buchmeier et al., 1995; Ibanez-Ruiz et al., 2000; Seaver and Imlay, 2001; Pardo-Este et al., 2018). Under oxidative stress and Mn^2+^ deplete conditions, KatN seems to be dispensable for *Salmonella* growth, likely reflecting redundancy of multiple peroxide degrading enzymes such as the alkyl/thiol hydroperoxide reductases AhpC and TsaA as well as peroxiredoxin Tpx (Hebrard et al., 2009; Horst et al., 2010; Diaz-Ochoa et al., 2016). Independently, H_2_O_2_-induced protein damage can be effectively repaired by thioredoxin and glutathione systems (Agbor et al., 2014; Song et al., 2016). The redundancy of antioxidant defenses in *Salmonella* attest to the tremendous selective pressure this intracellular pathogen faces during the respiratory burst of professional phagocytes.

### Cambialistic Enzymes

Because of the high binding affinity and ready availability in anoxic environments of the primitive Earth, Fe^2+^ was incorporated as cofactor of many primordial metabolic enzymes (Imlay, 2014). However, Fe^2+^ bound to a polypeptide can reduce H_2_O_2_, generating reactive hydroxyl and ferryl radicals *in situ* (Touati, 2000). This feature predisposes the Feα in [4Fe-4S] clusters of dehydratases and mononuclear Fe^2+^ to H_2_O_2_ attack, and Fe^2+^-mediated reduction of H_2_O_2_ in proximity to DNA inflicts genotoxicity (Winterbourn, 1995; Henle et al., 1999; Park and Imlay, 2003; Anjem and Imlay, 2012). Iron and manganese exist in two interchangeable redox forms, 2+ and 3+. Due to the symmetry of half-filled d5 electron shells, Mn^2+^ and Fe^3+^ (3d5) are more thermodynamically stable than Mn^3+^ (3d4) and Fe^2+^ (3d6) (Lingappa et al., 2019). The Mn^3+^/Mn^2+^ and Fe^3+^/Fe^2+^ redox couples have potentials of 1.51 and 0.77 V, respectively. Therefore, Mn^2+^ is less likely to donate electrons than Fe^2+^ (Guillemet-Fritsch et al., 2005). It is for this reason that, under most biological conditions, Mn^2+^ is less reactive than Fe^2+^ (Nealson and Myers, 1992). The thermodynamic stability of Mn^2+^, lower reactivity, and identical coordination geometry favor the mismetallation of Fe^2+^ by Mn^2+^. Replacement of Fe^2+^ with Mn^2+^ prevents oxidative damage of metalloenzymes (Emerson et al., 2008; Puri et al., 2010; Hood and Skaar, 2012). Accordingly, members of the Enterobacteriaceae shift from an iron- to a manganese-centric metabolism following oxidative stress (Anjem et al., 2009; Aguirre and Culotta, 2012). Examples of cambialistic enzymes include Rpe in the pentose phosphate pathway as discussed below. Incorporation of Mn^2+^ in place of Fe^2+^ allows metabolic flow during exposure to oxidative stress.

### Mn^2+^-Dependent Nonproteinaceous Antioxidants

Manganese ions render bacteria resistant to oxidative stress, even in the absence of Mn-SOD (Horsburgh et al., 2002). This protection may be mediated by the Mn^2+^-dependent degradation of O_2_•^-^ and H_2_O_2_ (Archibald and Fridovich, 1981, 1982; Berlett et al., 1990; Yocum and Pecoraro, 1999). Mn^2+^ reacts with O_2_•^-^ to form transient MnO^2+^, which converts to manganous phosphate, H_2_O_2_ and H_2_O (Barnese et al., 2012). In turn, Mn^2+^ disproportionates H_2_O_2_ to H_2_O and O_2_ (Stadtman et al., 1990).

## Mn^2+^-Driven Central Metabolism in *Salmonella* Virulence

Growth of *Salmonella* in host cells relies on a versatile metabolism. Relevant to this review, Mn^2+^ impacts glycolysis, reductive TCA and the pentose phosphate pathway in *Salmonella* sustaining oxidative stress.

### Metabolism of Mn^2+^ in Glycolysis and Reductive TCA During Oxidative Stress

**FIGURE 2:**
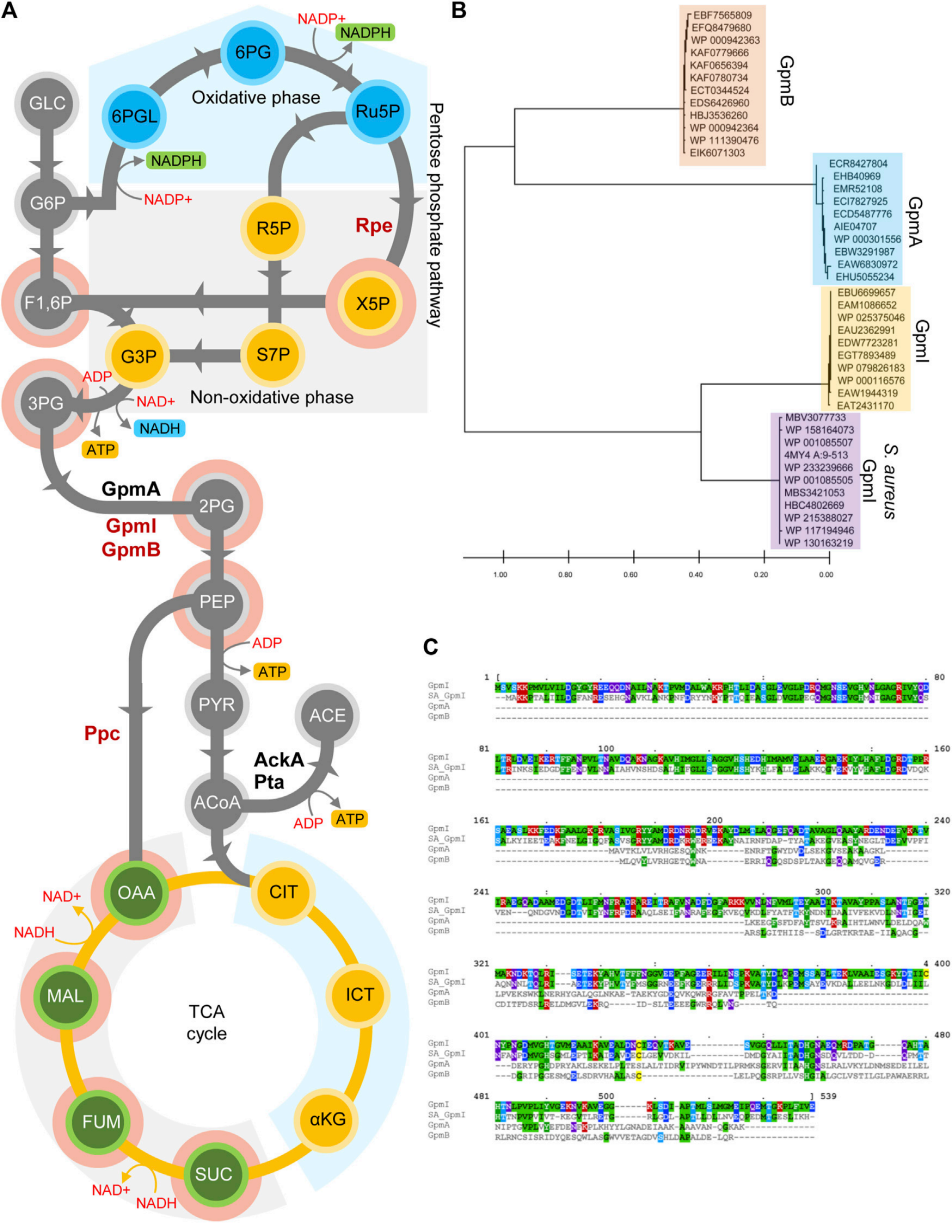
Manganese dependent metabolic adaptations in *Salmonella*. **(A)** Schematic representation of central metabolites and enzymes involved in glycolysis and TCA cycle. Enzymes in red are Mn^2+^ dependent. Glycolytic conversion of 3PG to 2PG is catalyzed by GpmA, a Mn^2+^-independent protein or its non-homologous isofunctional Mn^2+^-dependent GpmB and GpmI. During oxidative stress induced Mn^2+^ limitation, *Salmonella* utilizes the GpmA isoform to synthesise 2PG. The Mn^2+^-dependent phosphoenolpyruvate carboxylase (Ppc) shunts PEP into the reductive TCA cycle. Ribulose-5-PO_4_, 3-epimerase (Rpe), which catalyzes the conversion of ribulose-5PO4 to xylulose-5-PO4, is mismetallated during oxidative stress. As a result, *Salmonella* metabolism shifts into the production of reductive intermediates of the TCA cycle by Ppc and compromised non-oxidative phase of pentose phosphate pathway. **(B)** Phylogenetic analysis of *Salmonella* GpmA, B and I enzymes reveal that GpmI is similar to *S. aureus* GpmI. Differences between sequences are estimated by the scale shown at the bottom of the panel. **(C)** Clustal alignment of *Salmonella* GpmA (ACY87394.1), GpmB (ACY91834.1) and GpmI (ACY90848.1) with *S. aureus* GpmI (WP_001085507). Same scheme as in Figure 1D was followed to represent the alignment.

The electron transport chain is a source of ATP and a dominant pathway for balancing NADH/NAD^+^ redox. The oxidative inhibition of NDH-I NADH dehydrogenase in *Salmonella* undergoing oxidative stress decreases the energetic and redox outputs of the respiratory chain (Husain et al., 2008; Chakraborty et al., 2020). Thus, *Salmonella* experiencing oxidative stress favor glycolysis and fermentation (Figure 2A) to rescue ATP homeostasis and to balance redox. Glycolysis and associated fermentation generate ATP via substrate-level phosphorylation, produce intermediates for a variety of biosynthetic pathways, and balance redox (Figure 2A). Glycolysis is indispensable for the successful survival of *Salmonella* in host cells and is an essential component in resistance of *Salmonella* to the phagocyte NADPH oxidase (NOX2) (Bowden et al., 2009; Paterson et al., 2009; Götz and Goebel, 2010; Fitzsimmons L. et al., 2018; Chakraborty et al., 2020). *Salmonella* activate overflow metabolism in macrophages, partially to utilize the glycolytic products 3-phosphoglycerate (3PG) and 2-phosphoglycerate (2PG) as carbon sources (Jiang et al., 2021). Phosphoglycerate mutase (PGM) plays a unique role in controlling the overflow metabolism that mitigates oxidative stress in *Salmonella*. PGM, the third enzyme in the payoff phase of glycolysis, converts 3PG to 2PG. Many bacteria encode two analogous PGM enzymes with no sequence or structural similarity (Foster et al., 2010; Radin et al., 2019). The dPGM isoform utilizes the cofactor 2,3-bisphosphoglycerate, whereas the iPGM isoform requires Mn^2+^. While dPGM functions as a dimer or trimer, iPGM is active as a monomer (Foster et al., 2010). These *N*on-homologous *I S*ofunctional *E*nzymes (NISE) evolved independently to undertake the crucial metabolic conversion 3PG and 2PG, and bacteria may have accrued them via lateral gene transfer or non-orthologous gene displacement (Omelchenko et al., 2010). The genome of *Salmonella enterica* encodes two Mn^2+^-dependent iPGMs (GpmB and GpmI) and one Mn^2+^-independent dPGM (GpmA) (Figures 2B,C). The two Mn^2+^-dependent iPGMs are unique in sequence and structure. Our bioinformatic analysis revealed that *Salmonella* GpmB, which is structurally similar to *Bacillus* stearothermophilus phosphatase, PhoE, is conserved among major Enterobacteriaceae members, while two-domain monomer GpmI has around 50% sequence similarity with *Staphylococcus aureus* orthologue (Figure 2C), suggesting a common evolutionary origin (Figure 2B). Strikingly, *Salmonella* exposed to ROS produced in inflammation preferentially utilize the Mn^2+^-independent GpmA isoform over the Mn^2+^-cofactored GpmB enzyme (Chakraborty et al., 2020). The preferential utilization of the Mn^2+^-independent GpmA by *Salmonella* during resistance to NADPH oxidase-mediated host defense may be explained by the high demand for Mn^2+^ during periods of oxidative stress as hinted by the negative selection of *mntH* mutants after H_2_O_2_ treatment (Chakraborty et al., 2020). In addition to the constraints imposed by Mn^2+^ limitation, the NOX2-dependent acidification of the cytoplasm of intracellular *Salmonella* may also explain the preferential utilization of the acid phosphatase family member GpmA over its alkaline GpmB counterpart.

A knowledge-based and mathematical model of carbon flux in *Salmonella* revealed the importance of anaplerotic reactions around phosphoenolpyruvate (PEP) to oxaloacetate (OAA) conversion (Thiele et al., 2011; Dandekar et al., 2012). When modeled with glucose as sole C-source, the Mn^2+^-dependent PEP carboxylase enzyme (Ppc) seemed essential for fluxing glycolytic substrates into the reductive TCA cycle (Matsumura et al., 1999). *Salmonella* deficient of Ppc is virulent in BALB/c mouse model of infection (Tchawa Yimga et al., 2006). However, the combination of mutations in Ppc, acetate kinase (AckA) and phosphotransacetylase (Pta) results in a dramatic attenuation of *Salmonella* virulence (Chakraborty et al., 2020). Thus, generation of ATP via substrate-level phosphorylation together with the balancing of redox in the reductive TCA that is facilitated by fluxing PEP to oxaloacetate by the Mn^2+^-dependent Ppc contribute to *Salmonella* pathogenesis.

### Pentose-Phosphate Pathway

The mononuclear iron in ribulose-5-PO_4_, 3-epimerase (Rpe) in the pentose-phosphate pathway can be poisoned by submicromolar H_2_O_2_ concentrations (Sobota and Imlay, 2011). The inactive form of Rpe, however, can rapidly metallate with Mn^2+^ ions and revert to its active form (Sobota and Imlay, 2011). Metallation of Rpe with oxidative stress-resistant Mn^2+^ may allow for carbon flow through the pentose phosphate pathway, thereby generating NADPH reducing power that is needed to maintain antioxidant defenses such as glutathione or thioredoxin reductase (Song et al., 2016).

## Mn^2+^-Based Antinitrosative Defenses

Reactive nitrogen species synthesized by inducible nitric oxide (NO) synthase are bacteriostatic against *Salmonella* (Vazquez-Torres et al., 2000a; Thiele et al., 2011; Henard and Vázquez-Torres, 2012; Fitzsimmons L. F. et al., 2018). RNS modify biomolecules containing radicals, heme prosthetic groups, mononuclear iron, [Fe-S] clusters or redox active thiols in cysteine residues (Mikkelsen and Wardman, 2003; Forman et al., 2004; Poole, 2005; Husain et al., 2008; Pearce et al., 2009; Crawford et al., 2016; Jones-Carson et al., 2016; Jones-Carson et al., 2020). *Salmonella* mutants lacking Mn^2+^ transporters are more sensitive to RNS (Frawley et al., 2018; Yousuf et al., 2020; Ouyang et al., 2022). The intracellular concentrations of Mn^2+^ increase in *Salmonella* undergoing nitrosative stress, likely reflecting the upregulation of Mn^2+^ importers (Richardson et al., 2011; Yousuf et al., 2020). Regulation of Mn^2+^ transport systems is under the control of the transcription factor DksA (Crawford et al., 2016). Maintaining the homeostasis of intracellular Mn^2+^ in *Salmonella* after exposure to NO also involves the MntP and FieF efflux pumps (Ouyang et al., 2022). It remains unknown if these Mn^2+^ efflux systems contribute to the antinitrosative defenses of *Salmonella*.

Transient drops in intracellular amino acids during NO stress induce RelA-catalyzed synthesis of the (p)ppGpp alarmone, and the hydrolytic activity of SpoT is essential for reestablishing ppGpp homeostasis (Richardson et al., 2011; Fitzsimmons L. F. et al., 2018). A Mn^2+^ ion is coordinated by at least two carboxylates from aspartate and glutamate residues in the hydrolase domain of SpoT (Hogg et al., 2004). Histidine along with H_2_O molecules coordinate the rest of the four electrons of Mn^2+^. Studies in *E. coli* and *Streptococcus* revealed that ppGpp hydrolysis is strictly dependent on Mn^2+^ ions (Heinemeyer et al., 1978; Mechold et al., 1996). The nucleotidyltransferase domain (cd05399) of SpoT has a metal binding region dominated by aspartate and glutamate residues. Interestingly, the aspartate and glutamic acid residues of CD05399 are conserved in the synthetase domain rather than the hydrolase domain of SpoT, raising the possibility that Mn^2+^ may catalyze ppGpp synthesis rather than hydrolysis in *Salmonella’*s SpoT proteins. However, studies in *Streptococcus* revealed that intramolecular signal transmission between the two domains in the presence of ppGpp creates an allosteric shift in the synthetase domain, resulting in coordination of Mn^2+^ by an additional aspartate (Hogg et al., 2004). The additional coordination suppresses synthetase enzymatic activity. Interestingly, intracellular *Salmonella* lacking the non-catalytic regulatory C-terminal domain of SpoT (i.e., SpoT-ΔCTD) transcribe abnormally low levels of the Mn^2+^ importer SitABCD in macrophages (Fitzsimmons et al., 2020). SpoT-ΔCTD-expressing *Salmonella* are attenuated in NRAMP1^+^ C3H/HeN mice but not in NRAMP1^-^ C57BL/6 mice, suggesting that the SpoT-dependent regulation of SitABCD combats the Mn^2+^ restrictions associated with a functional NRAMP1 transporter (Fitzsimmons et al., 2020).

## Conclusion

Mn^2+^ homeostasis plays a poorly understood, but vital role in *Salmonella* pathogenesis. The elaborate transcriptional and posttranscriptional regulation of expression of diverse Mn^2+^ uptake and efflux systems help *Salmonella* navigate different metal-restricted anatomical sites in the vertebrate host. Historically, Mn^2+^ has been recognized as a cofactor of critical antioxidant defenses of *Salmonella*. Mn^2+^-dependent SOD protects *Salmonella* from oxidative stress; a function for Mn^2+^-dependent catalase in *Salmonella* virulence remains to be determined. Recent investigations have revealed the intricate relations between Mn^2+^ homeostasis, central metabolism, and antioxidant defenses of *Salmonella*. *Salmonella* rely on Mn^2+^ independent glycolysis during their adaptations to oxidative killing, but use Mn^2+^ to power anapleurotic pentose phosphate pathway reactions involved in redox balance necessary for central metabolism and synthesis of reductive power that fuels classical antioxidant defenses, such as glutathione reductase. Many unanswered questions still exist about the involvement of Mn^2+^ in the adaptations that promote *Salmonella* pathogenesis, providing a myriad of opportunities for future research.
